# An Outbreak of Dengue Virus Serotype 2 Cosmopolitan Genotype in Nepal, 2017

**DOI:** 10.3390/v13081444

**Published:** 2021-07-24

**Authors:** Mya Myat Ngwe Tun, Kishor Pandey, Takeshi Nabeshima, Aung Kyaw Kyaw, Mandira Adhikari, Sandra Kendra Raini, Shingo Inoue, Shyam Prakash Dumre, Basu Dev Pandey, Kouichi Morita

**Affiliations:** 1 Department of Virology, Institute of Tropical Medicine, Nagasaki University, Nagasaki 852-8523, Japan; myamyat@tm.nagasaki-u.ac.jp (M.M.N.T.); mtmikami@tm.nagasaki-u.ac.jp (T.N.); akkyawdmr@gmail.com (A.K.K.); kendraraini@gmail.com (S.K.R.); pampanga@nagasaki-u.ac.jp (S.I.); 2 Central Department of Zoology, Institute of Science and Technology, Tribhuvan University, Kathmandu 44060, Nepal; drkishorpandey1@gmail.com; 3 Shi-Gan International College of Science and Technology, Kathmandu 44060, Nepal; mandiraadhikari.ma@gmail.com; 4 Central Department of Microbiology, Institute of Science and Technology, Tribhuvan University, Kirtipur 44618, Nepal; sp.dumre@gmail.com; 5 Ministry of Health and Population, Kathmandu 44060, Nepal

**Keywords:** dengue, dengue virus serotype 2, cosmopolitan genotype, whole genome sequencing, Nepal

## Abstract

Dengue virus (DENV) is one of the most prevalent neglected tropical diseases, with half of the world’s population at risk of infection. In Nepal, DENV was first reported in 2004, and its prevalence is increasing every year. The present study aimed to obtain and characterize the full-length genome sequence of DENV from the 2017 outbreak. Hospital-based surveillance was conducted in two provinces of Nepal during the outbreak. Acute-phase serum samples were collected from 141 clinically suspected dengue patients after the rainy season. By serological and molecular techniques, 37 (26.9%) and 49 (34.8%), respectively, were confirmed as dengue patients. The cosmopolitan genotype of DENV-2 was isolated from 27 laboratory-confirmed dengue patients. Genomic analysis showed many amino acid substitutions distributed mainly among the E, NS3, and NS5 genes. Phylogenetic analyses of the whole genome sequence revealed two clades (Asian and Indian) among DENV-2 isolates from Nepal. The DENV isolates from hilly and Terai areas were similar to Asian and Indian strains, respectively. Further genomic study on different DENV serotypes is warranted to understand DENV epidemics in Nepal, where there are limited scientific resources and infrastructure.

## 1. Introduction

Dengue virus (DENV) is an emerging mosquito-borne arboviral infection endemic in urban and suburban areas of tropical and subtropical countries globally [1]. It is transmitted to humans by the bite of infected female mosquitoes, mainly *Aedes aegypti* and *A. albopictus*. DENV causes a wide spectrum of disease severity, ranging from flu-like illness (dengue with and without warning signs) to life-threatening conditions known as severe dengue [2]. The four serotypes of DENV (DENV1, DENV-2, DENV3, and DENV4), which belong to the genus Flavivirus of the family Flaviviridae, are antigenically and genetically distinct. Infection with one of these DENV serotypes provides lifelong immunity to that particular serotype only [3]. When the same person obtains a second DENV infection from a different serotype, it can lead to severe dengue with dengue hemorrhagic fever/dengue shock syndrome [4]. Globally, about 1% of dengue-infected people die of this disease if it is not managed properly [5].

DENV is a single-stranded, positive-sense RNA virus with a genome length of approximately 11 kb [6]. Its genome has a long, open reading frame that encodes a polyprotein and is flanked by the 5′- and 3′-untranslated regions. It encodes three structural proteins (capsid (C), membrane (M) and envelope (E)) and seven nonstructural (NS) proteins (NS1, NS2A, NS2B, NS3, NS4A, NS4B, and NS5) [6]. The major role of the structural proteins is to contribute to the pathogenic function of the virus, mainly flexibility in host attachment, virulence, and replication [7]. The nonstructural proteins could play important roles in the evolution of DENV in hyperendemic areas. Increasing severity and transmission are known to be associated with amino acid variations in nonstructural DENV proteins [8]. Complete genome sequencing has significantly improved our understanding of the full extent of the genomic diversity of DENV and the consequences of such genetic variation in functional terms. DENV serotypes are further classified into genotypes and lineages based on the nucleotide sequences due to the high mutation rates [9].

It is estimated that among 390 million dengue infections reported every year worldwide, 96 million manifest clinically with any severity of dengue, with some requiring hospitalization [5]. The worldwide incidence of dengue has increased eightfold over the last 2 decades (0.5 million in 2000 to 4.2 million in 2019) [5]. Around 1.8 billion people in the WHO South-East Asia and Western Pacific regions are at risk of dengue. During the last few decades, DENV has emerged in South Asia, and epidemics of varied magnitude have occurred in Bhutan, Nepal, India, Maldives, Bangladesh, and Pakistan [10].

DENV is an emerging disease in Nepal and was first reported in 2004 [11]. After this first report, several outbreaks occurred in different areas of Nepal, where all four serotypes were reported [12,13,14]. Dengue-epidemic outbreaks occur at 2- or 3-year intervals, with one dominant serotype varying over time. The number of reported dengue patients has been increasing every year, and the largest outbreak was in 2019 [15,16]. Virus evolution (genotype or serotype shifting) is an important factor in causing outbreaks [17]. Few whole genomes of the DENV strains circulating in Nepal have been sequenced. To understand the virus’s evolution, the molecular study of circulating DENV serotypes and genotypes during epidemics is essential. The present study aimed to understand the molecular epidemiology of DENV in different geographical areas in Nepal during a large outbreak that exploded after heavy rainfall and flooding in 2017.

## 2. Materials and Methods

### 2.1. Study Design and Sites

**Figure 1:**
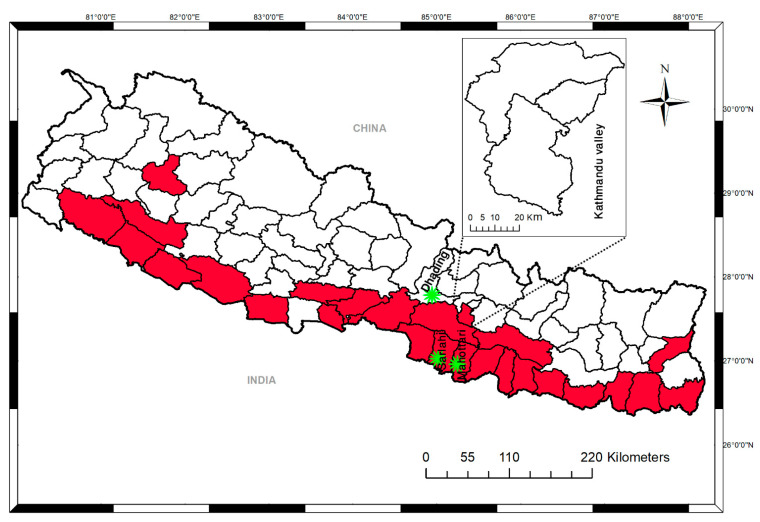
District-level map of Nepal showing the study sampling sites and areas affected by flooding and landslides in 2017. Areas in red indicate the districts affected. Asterisks indicate the sampling sites.

This cross-sectional study was carried out in November 2017. During the rainy season, heavy rainfall had resulted in flooding and landslides, affecting 26 districts of Nepal. After a month, there was an outbreak of febrile illness. The present study was conducted in two areas (lowland Terai and hilly) of Nepal. Mahottari and Sarlahi represented the lowland Terai region of Nepal, and Dhading represented the hilly area. These two districts are known to report DENV cases almost every year. A total of 141 patients with the presumptive diagnosis of dengue on rapid diagnostic testing (119 antibody detections and 22 NS1 detections) were included. Serum samples were collected from primary healthcare centers of Lalbandi, Sarlahi, and Bardibas of Mahottari (lowland Terai) and Dhading District Hospital, in the hilly region of Bagmati Province (Figure 1). Sarlahi and Mahottari are near the border with India, where the temperature is usually high. Mean annual rainfall and seasonal temperatures greatly vary in the two ecologically distinct sampling sites (Terai and hilly areas).

### 2.2. Sample Collection

Blood samples were collected from individuals with a febrile illness clinically resembling DENV infection. Basic demographic information (age and sex) and clinical information were obtained with a questionnaire using direct interviews. A blood sample (3 mL) was collected from each patient and allowed to clot at room temperature. The samples were centrifuged to separate serum, which was stored at −20 °C until further study. Serum samples were used for NS1-antigen detection using an SD Bioline rapid test (Standard Diagnostic, Seoul, Korea). Serological and molecular studies were performed at the Department of Virology, Institute of Tropical Medicine, Nagasaki University, Japan.

### 2.3. Serological Examination (Anti-DENV IgM and Anti-DENV IgG)

Serological examination of DENV infection was performed by an in-house anti-DENV IgM antibody capture (MAC) ELISA and anti-DENV IgG indirect ELISA. In-house MAC ELISA was performed to confirm acute DENV infection, as described previously [18,19]. Optical density (OD) was read at 492 nm, and a P:N (positive-control OD or sample OD/negative control OD) ratio ≥ 2 was considered positive. In-house DENV IgG indirect ELISA [20] was used to determine primary and secondary infection and had a high correlation with dengue hemagglutination inhibition, the gold standard recommended by the WHO [1]. A sample titer ≥ 1:3000 (cutoff value for positive IgG was 1:1000 ± 3 standard deviations) was considered DENV IgG-positive. If the IgG titer were ≥29,000, infection was considered secondary dengue, whereas a titer *<* 29,000 was considered primary dengue [20].

### 2.4. Virus Isolation in Cell Culture 

Serum samples (10 µL) were inoculated into *A. albopictus* clone mosquito cells (C6/36 E2) grown in flat culture tubes to isolate DENV. Virus-infected cells were incubated at 28 °C for 7 days in Eagle’s minimum essential medium supplemented with 2% fetal calf serum and 0.2 mM non-essential amino acids [21,22]. Infected culture fluid (ICF) was collected from each tube, aliquoted, and stored at −80 °C until further use. A second virus passage was completed for 1 week in tubes with fresh confluent cells with the same incubation conditions as the first passage. ICF was collected and processed from each tube for further molecular analysis.

### 2.5. RNA Extraction and Conventional Reverse Transcription (RT)-PCR 

RNA was extracted from 140 μL ICFs for the presence of DENV using a viral RNA Mini Kit (Qiagen, Hilden, Germany) according to the manufacturer’s protocol. The presence of DENV RNA was confirmed by RT-PCR protocol using a PrimeScript One-Step RT-PCR Kit version 2 (Takara Bio, Shiga, Japan) following the manufacturer’s protocol. RT-PCR amplification was carried out in a final volume of 25 μL with 5 μL RNA. The PCR mixture consisted of 1 μL enzyme mix, 13 μL 2× buffer, 4 μL nuclease-free water, 1 μL of 100 pmol forward and reverse primers, with separate primer sets for the detection of DENV and determination of specific DENV serotypes [22,23,24].

### 2.6. Quantification of DENV Genome Levels by Real-Time RT-PCR 

Viral RNA was extracted from 140 μL of serum sample using the same kit used for RNA extraction from ICF. RNA (5 μL) was used for quantitative real-time RT-PCR (qRT-PCR). The envelope (E) gene was targeted for amplification in 20 μL total reaction mixture consisting of 5 μL TaqMan Master Mix, 9 μL nuclease-free water, 0.3 μL of 100 pmol forward and reverse primers, and 0.4 μL probe with DENV serotype-specific primers of TaqMan Fast Virus 1-Step Master Mix (Life Technologies, CA, USA) following a protocol described previously [25]. Viral genome levels are expressed as log_10_ genome copies/mL.

### 2.7. Whole-Genome Sequencing by Next-Generation Sequencing (NGS) and Phylogenetic Tree Analysis 

Whole-transcriptome libraries (Ion Total RNA-Seq Kit version 2, Life Technologies, Carlsbad, CA, USA) were synthesized using RNA extracted from ICF. Sequencing was performed using NGS (Ion Proton, Life Technologies, Carlsbad, CA, USA). Low-quality reads of <75% with a quality score *<* 20 were removed from the input data file using the FastX Toolkit 0.0.14. Sequence quality was checked before and after quality trimming by FastQC 0.11.8. For de novo assembly, Trinity 2.8.4 [26] was used, and the sequence name was repaired by SeqKit v 10.0.1. BLASTn 2.7.1 [27] was used to assemble the de novo contig. The trimmed FastQ data set was mapped using BWA 0.7.17 [28] to the reference sequence chosen by BLASTn, and variants were detected by LoFreq 2.1.3.1 [28] and VarScan 2.4.3 [28]. From the VarScan output, Samtools 1.9 constructed the consensus DENV sequence. Data preprocessing was conducted according to the best-practice workflow for GATK 3.8.1 and Picard 2.20. DENV sequences in the International Nucleotide Sequence Database Consortium were collected and annotated with Entrez Direct and annotated by SeqKit. Sequences of the full-genome coding region were aligned by MAFFT 7.407 [29]. Maximum-likelihood phylogenetic trees were constructed by PhyML 3.2.0 [30]. Bootstrap values were obtained after 1000 replications. The substitution model was selected by jModelTest 2.1.10 [31].

### 2.8. Statistical Analysis

All data on patients’ clinical and laboratory parameters were transcribed into a spreadsheet (MS Excel), and appropriate data verification and cleaning were performed. Data were analyzed using SPSS version 22.0. Categorical variables were presented as absolute number (n) and percentage (%) as appropriate. Continuous variables (ratio of nonsynonymous to synonymous mutations) were compared using the Mann–Whitney U test. α = 0.05 was used for all statistical analyses, and *p* < 0.05 was considered statistically significant.

## 3. Results

### 3.1. Demographics and Clinical Findings

After the first dengue report in 2004, major dengue outbreaks occurred in 2006, 2010, 2013, and 2016 with 32, 917, 683, and 1527 cases, respectively. The overall trend of dengue cases in Nepal shows a clear increase over the period. Heavy rainfall that started in August 2017 resulted in massive flooding and landslides, affecting 26 districts in the Terai region (Figure 1). The number of febrile patients rapidly increased in the affected districts. A total of 141 blood samples were collected from Sarlahai and Mahottari (n = 107) and Dhading district (n = 34), where flooding and landslides had occurred.

**Figure 2:**
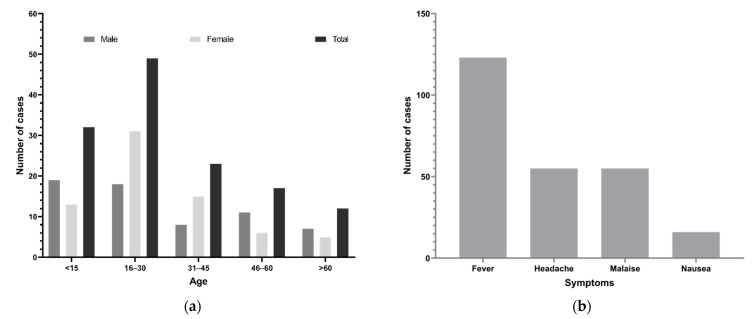
Demographics (age, sex) and clinical features of dengue suspected patients during the 2017 outbreak, Nepal. (**a**) Number of dengue suspected cases by age and sex groups. (**b**) Common symptoms.

The age range of patients with suspected dengue was 4 months to 76 years (median 27 years). The highest number of cases (49, 34.8%) were aged 16–30 years (Figure 2a), followed by <15 years (32, 22.7%). The most common clinical features of dengue-suspected cases confirmed by rapid diagnostic tests were fever (97%, 123/127), headache (43%, 55/127), malaise (43%, 55/127), coughing (38%, 31/81), vomiting (24%, 19/81), eye pain (14%, 11/81), and nausea (13%, 16/127; Figure 2b). Note that patients whose clinical information was missing were excluded during the analysis of clinical features.

**Figure 3:**
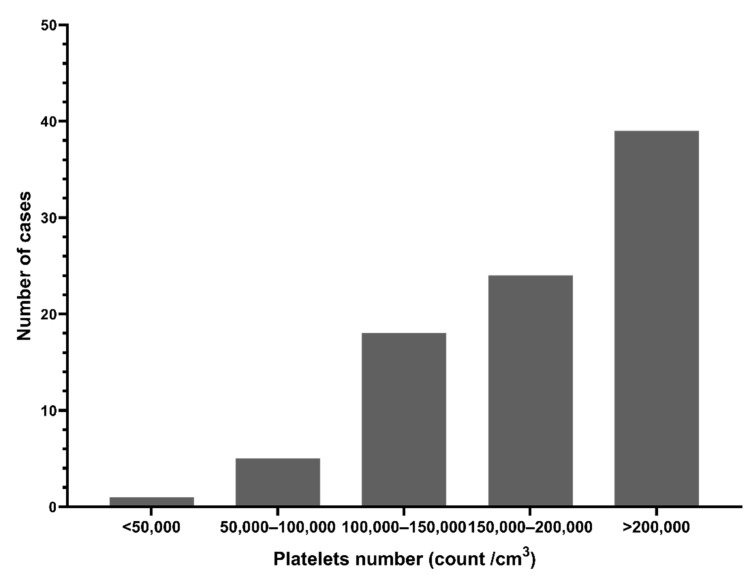
Platelet counts in dengue suspected patients during the 2017 outbreak, Nepal.

Platelet counts were below the normal range (median 190,000/cm^3^) Figure 3 shows 27.6% (24/87) of patients had platelet counts < 150,000/cm^3^, and 72.4% (63/87) had counts > 150,000/cm^3^ (n = 87). Hemoglobin levels and total WBC counts were within the normal range (data not shown).

### 3.2. Serological Findings

A total of 141 serum samples were investigated using MAC ELISA for DENV, and 22.0% (31/141) were found to be positive. Those samples were also used for DENV IgG indirect ELISA, with 18.4% (26/141) positive and 81.6% (115/141) negative. Among DENV IgG-positive patients, 7 (5.0%) and 19 (13.4%) had a primary and secondary dengue infection, respectively. Among the 141 patients, 19 (13.5%) tested positive for both DENV IgM and IgG antibodies.

### 3.3. Molecular Findings

All serum samples were tested for DENV by qRT-PCR, and the results showed that 34.8% (49/141) were positive for the DENV-2 serotype. Of these, virus isolation was successful in 27 samples, confirmed by conventional RT-PCR (Table 1). Mean serum-viremia levels were 5.2 (log_10_ genome copies/mL) in the 49 real-time PCR-positive patients and 6.4 in 27 patients with isolated DENV, respectively (Supplementary Figure S1).

### 3.4. Phylogenetic Analysis

**Figure 4:**
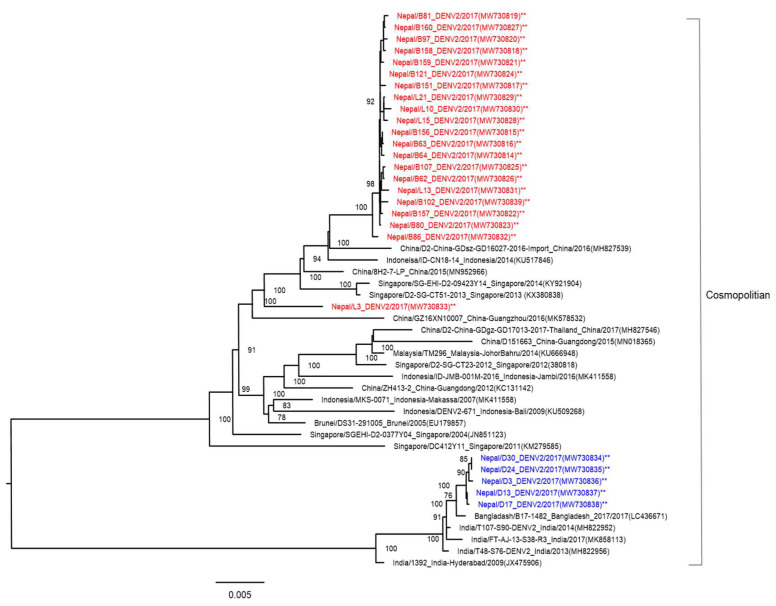
Phylogenetic tree based on whole-genome sequencing of 26 DENV-2 strains (indicated by asterisks, and red and blue colored fonts for each clade) isolated from the 2017 dengue outbreak in Nepal. The DENV-2 phylogenetic tree was reconstructed using the maximum likelihood method. The 20 reference strains used in the phylogenetic tree were obtained from Genbank and are named by country origin, strain name, year of isolation, and GenBank accession number.

NGS analysis was performed, and the complete DENV-2 genome was determined from the ICF of cells inoculated with the 26 patients’ serum samples. One sample (L-40) was not read by NGS. To compare the genetic background of DENV-2 strains, we compared the sequences of the DENV-2 isolates from this study with reference sequences of DENV-2. We examined the genetic relationship of the present 26 isolates and the other 22 DENV-2 strains isolated from diverse geographical areas and obtained from GenBank by performing phylogenetic analysis on their complete genome sequences. All were the DENV-2 cosmopolitan genotype (Figure 4). DENV-2 isolates from Nepal showed two clades (Asian and Indian). The 20 DENV-2 isolates derived from hilly areas of Nepal belonged to Asian strains (similar to Chinese, Indonesian, and Singaporean strains), while five isolates belonging to Indian strains were derived from Terai, which borders India.

**Figure 5:**
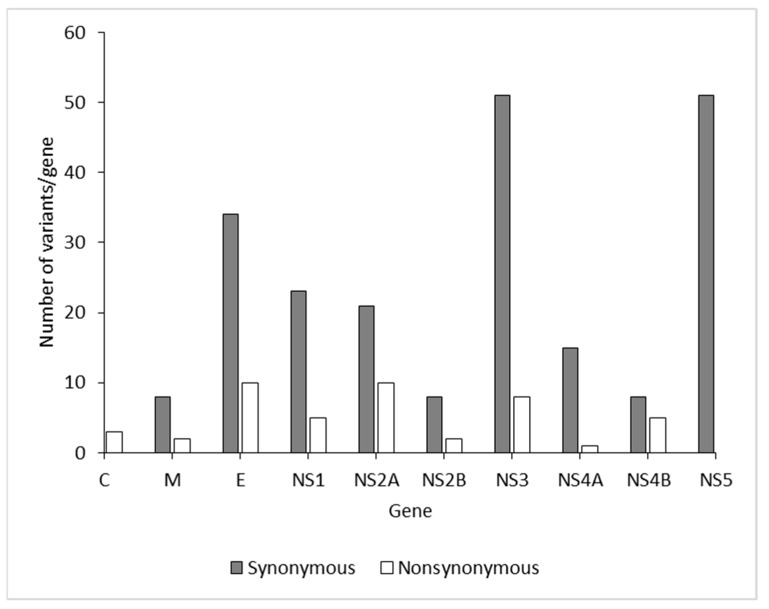
Positions with variant incidence > 1% per gene among the DENV-2 isolates from the 2017 outbreak in Nepal.

To characterize viral amino acid differences using NGS, the amino acid sequences of the DENV-2 isolates were aligned, which revealed 99% shared identity among the isolates. Compared with 26 isolates, amino acid substitutions were identified in 165 sites throughout the complete coding region of the five DENV-2 isolates in this study (Table 2). Across the complete genome, the number of positions with variant frequency *>* 1% revealed 223 synonymous and 58 nonsynonymous mutation sites among DENV isolates in the present study (Figure 5). There was no difference in the median mutation ratio of synonymous to nonsynonymous mutation sites between dengue structural (median 3.4, IQR 2.36–3.7) and nonstructural proteins (median 4.25, IQR 3.1–5.5; *p* = 0.183 (Mann–Whitney *U* test); Supplementary Figure S2). The major amino acid substitutions were distributed among the E, NS3, and NS5 genes. Variant frequency *>* 50% indicated that these variants had become predominant in the particular patient (Table 2).

## 4. Discussion

Dengue has been identified as one of the important emerging infectious diseases in Nepal. Establishing a molecular database is very important and necessary to understand DENV evolution, virulence, and spread. This information is lacking in Nepal. This report describes the basic molecular database of DENV variants isolated from Nepal. The first case in Nepal was identified in 2004 [11]. DENV epidemics occur during and after the rainy season in Nepal [32,33]. Several factors related to mosquito invasion and increased travel among the populace could be drivers of the expanding epidemic and could explain the introduction of DENV to newer locations. Population growth has led to unplanned and uncontrolled urbanization, which has caused deterioration of the environment, water, sewage, and waste management in many parts of the country. The crowded human population living in close contact with increasingly higher-density mosquito populations creates suitable conditions for increased dengue infection.

The age distribution of suspected clinical dengue cases showed that the highest number of cases were aged 15–30 years (34.8%), followed by those aged < 15 years (22.7%). This result is similar to data obtained in the 2016 outbreak, in which dengue-positive cases were recorded more in those aged > 15 years [14,15,34]. Seropositivity is generally higher in younger people (age 15–30 years) because they are more active in outdoor activities and are likely to be more exposed to mosquitoes. In the 2017 outbreak, common clinical features were fever, headache, myalgia, and nausea. Relatively less common clinical features were retro-orbital pain, anorexia, arthralgia, skin rash, and abdominal pain. The severity of these clinical features was different in each patient, depending upon the DENV serotype and host immune systems. Similar results have been obtained in previous studies [15,34]. However, skin rash, retro-orbital pain, and hemorrhagic manifestations are more specific than other features in dengue cases.

In the present study, 56.7% (80/141) of cases were confirmed as dengue infection by DENV IgM ELISA, DENV IgG ELISA, and DENV real-time RT-PCR. The 43% (63/141) of patients detected as recent dengue infection were confirmed by DENV IgM ELISA and DENV real-time RT-PCR. The remaining dengue-negative cases might have been due to other febrile illnesses, such as chikungunya fever, influenza, Japanese encephalitis, measles, typhoid, rickettsiosis, and malaria [19]. It is also possible that the antibody against dengue had not yet been produced by patients when the serum was collected during the acute phase of illness. Convalescent-phase serum samples were not available in this study. The 18% of secondary infections found in this study may relate to patients presenting with febrile illness and not severe symptoms. In a previous study, there was a higher prevalence of secondary DENV infection than primary infection [14,35,36]. Relevant authorities should work toward preparedness for future outbreaks.

Four dengue serotypes were reported in 2006 and 2010 [14,37]. The dominant serotypes in the dengue outbreaks of 2010, 2013, and 2016 were DENV-1,-2, DENV-2, and DENV-1, respectively. Here, we confirmed that the DENV-2 cosmopolitan genotype was responsible for the 2017 outbreak. This genotype spread widely throughout Sri Lanka, India, Bangladesh, China, Thailand, Reunion Islands, Israel, Seychelles, and the Indian Ocean islands in 2017 [22,38,39,40,41,42]. In contrast to serotypes, very little is known about the DENV genotypes circulating in Nepal. The Terai region of Nepal borders India, and hence it is expected that DENV strains isolated from Nepal originated in India. The phylogenetic tree showed that the DENV-2 isolated in Nepal during the 2017 outbreak likely originated in India; however, this was not true for all of the DENV-2 isolates in this study (Figure 4). The phylogenetic tree showed that 20 of the DENV-2 isolates were genetically close to other Asian DENV-2 isolates, mainly from China (2015 and 2016), Indonesia (2014), and Singapore (2013 and 2014). The largest number of international tourists arriving in Nepal in 2017 were from India (155,784), China (108,839), the US (79,255), the UK (52,690), and Sri Lanka (45,313) [43]. There was a strong relationship between the number of international travelers and DENV-2 strains isolated in Nepal. These results also showed that different DENV strains found in Nepal were imported from different geographical areas before there was local transmission. Similar to the present study, DENV strains were imported from different geographical areas in studies reported in China, Singapore, and Vietnam [44]. The diversity of DENV-2 strains has been shown to play an important role in the resurgence of severe dengue infection [45].

In the whole-genome sequences of DENV-2 strains from Nepal, 26 strains showed many amino acid substitutions (223 synonymous and 53 nonsynonymous) in structural and nonstructural proteins (Table 2). Our study found many mutations (34 synonymous and 10 nonsynonymous) in the E gene. A change in amino acids in the E gene may enhance neutralization during pathogenesis. In addition to the E gene, amino acid variants in several positions were observed in the NS1 (n = 5), NS2A (n = 10), NS2B (n = 2), NS3 (n = 8), NS4A (n = 1), NS4B (n = 5), and NS5 (n = 12) genes. Interestingly, some amino acid substitutions (E203G and K1047R) observed in the M and NS1 proteins in this study had also been reported previously [46,47]. These substitutions may potentially influence viral fitness and virulence in host cells. These variations in the DENV genome sequence are probably due to selection pressure, adaptive evolution, and ecology [48].

## 5. Conclusions

We report the isolation and complete genome analysis of DENV-2 strains isolated from serum samples during the 2017 outbreak in Nepal. The whole-genome sequences of 26 DENV-2 isolates from Nepal with amino acid variations were compared with other DENV-2 strains circulating in different parts of the world to determine the genetic relationships. We concluded that genetic variations were quite high among the envelope gene of DENV-2 in Nepal during 2017. These data show the pathogenic characteristics of DENV-2 and their role in dengue outbreaks in Nepal. These findings are very important for preparedness in preventing future outbreaks and can both guide national governments in forming public-health policies and boost the efficiency of an early warning system for new viral strains. Moreover, continued monitoring of full-length genomic information of DENV from a model country such as Nepal with extensive altitudinal and climatic variations may explore whether such variations contribute to the genetic makeup of DENV as a consequence of adaptation. These types of genomic monitoring are helpful in executing timely outbreak interventions, and these sequence databases contribute to understanding the dynamics of dengue evolution at national and global levels. Most importantly, these data will be essential in monitoring outbreak trends and establishing predictive models for early outbreak detection.

## Figures and Tables

**Table 1:** Demographic and laboratory profiles of dengue suspected patients with positive DENV isolation results (*n* = 27).

Sample No.	Patient Code	Location	Age	Sex	Days of Fever	IgM Ratio	IgG Titer	Serum Viremia Level (log_10_ Copies/mL)	Virus Isolation Serotype
1	B-102	Bardibas	12	M	2	0.9	178	8.0	DENV-2
2	B-107	Bardibas	62	M	2	0.7	324	7.0	DENV-2
3	B-57	Bardibas	5	F	N/A	1.0	282	7.6	DENV-2
4	B-151	Bardibas	36	F	N/A	**2.3**	**13,036**	5.7	DENV-2
5	B-156	Bardibas	N/A	N/A	N/A	**5.0**	1702	4.9	DENV-2
6	B-157	Bardibas	N/A	N/A	N/A	0.8	1674	5.7	DENV-2
7	B-158	Bardibas	N/A	N/A	N/A	1.0	278	5.9	DENV-2
8	B-159	Bardibas	N/A	N/A	N/A	1.7	203	4.9	DENV-2
9	B-160	Bardibas	N/A	N/A	N/A	**3.2**	117	9.2	DENV-2
10	B-62	Bardibas	35	M	N/A	0.7	212	6.8	DENV-2
11	B-63	Bardibas	20	M	N/A	1.0	404	7.5	DENV-2
12	B-64	Bardibas	32	F	N/A	0.7	849	7.6	DENV-2
13	B-80	Bardibas	22	F	3	1.5	223	5.6	DENV-2
14	B-81	Bardibas	22	M	2	0.8	200	7.3	DENV-2
15	B-86	Bardibas	50	F	N/A	**6.5**	**6112**	7.3	DENV-2
16	B-97	Bardibas	48	M	2	0.9	395	7.4	DENV-2
17	D-13	Nilkantha	40	M	5	0.2	217	8.7	DENV-2
18	D-17	Nilkantha	21	M	2	0.6	219	6.1	DENV-2
19	D-24	Nilkantha	42	F	3	0.5	305	4.1	DENV-2
20	D-3	Nilkantha	15	F	3	**2.2**	135	4.5	DENV-2
21	D-30	Nilkantha	20	F	3	0.6	409	5.0	DENV-2
22	D-40	Nilkantha	30	F	3	0.3	342	5.8	DENV-2
23	L-10	Lalbandi	46	F	5	3.9	997	5.5	DENV-2
24	L-13	Hariwon	26	M	7	**2.9**	**23,375**	5.5	DENV-2
25	L-15	Lalbandi	30	F	3	**6.8**	**9629**	7.7	DENV-2
26	L-21	Lalbandi	18	M	5	9.4	221	5.5	DENV-2
27	L-3	Lalbandi	30	F	5	**25.6**	**28,016**	7.0	DENV-2

N/A = not available; M = male; F = female; bold font indicates that IgM positive cutoff ≥ 2, and IgG positive cutoff ≥ 3000.

**Table 2:** Non synonymous variants (≥1%) alleles shared among the complete genome of DENV-2 isolates from 2017 outbreak, Nepal.

Sample No.	Feature	Nucleotide Position	Amino Acid Position	Reference Allele	Alternate Allele	Frequency (%)	Amino Acid Change
20	C	418	108	C	A	7	L-M
20	C	431	112	C	T	7	A-V
20	C	435	113	A	G	7	I-M
1, 2, 3, 4, 5, 6, 7, 8, 9, 11, 13, 14, 17, 18, 19, 23, 24, 25, 26	M	704	203	A	G	22–62	E-G
21	M	817	241	A	G	1	I-V
23	E	954	286	A	G	52	I-M
17, 19	E	1046	317	C	A	28–55	T-N
9	E	1481	462	C	T	5	T-M
27	E	1649	518	T	A	1	V-D
12	E	1688	531	T	C	14	V-A
12	E	1691	532	T	C	13	V-A
27	E	1960	622	T	A	1	L-M
5, 6, 9, 10, 12, 14, 16, 24, 25, 26	E	1964	623	A	G	22–100	E-G
26	E	2009	638	T	C	13	V-A
20	E	2386	764	G	A	6	V-I
20	NS1	2452	786	A	G	1	K-E
15	NS1	2482	796	A	G	1	I-V
16	NS1	2726	877	G	A	6	R-Q
20	NS1	3236	1047	A	G	7	K-R
20	NS1	3289	1065	A	G	12	N-D
21, 20	NS2A	3574	1160	T	A	20–31	L-I
21, 20	NS2A	3598	1168	A	G	36–37	M-V
20	NS2A	3598	1168	G	A	36	V-M
20	NS2A	3664	1190	G	A	22	A-T
9	NS2A	3736	1214	T	C	6	F-L
12	NS2A	3799	1235	A	G	10	I-V
23	NS2A	3803	1236	T	C	16	V-A
4	NS2A	3886	1264	G	A	100	V-I
19	NS2A	4126	1344	A	G	1	K-E
20	NS2A	4127	1344	A	G	1	K-R
21	NS2B	4387	1431	A	G	1	I-V
15	NS2B	4439	1448	T	G	1	L-R
20	NS3	4612	1506	C	T	11	L-F
11, 17, 21	NS3	4857	1587	A	C	1–21	K-N
11, 17, 19, 21	NS3	4859	1588	C	A	1–17	P-H
15	NS3	4954	1620	A	G	1	K-E
8	NS3	5032	1646	G	A	97	G-S
20	NS3	5078	1661	G	A	13	R-K
20	NS3	5902	1936	A	G	5	I-V
27	NS3	6071	1992	A	G	50	D-G
10, 12, 23	NS4A	6712	2206	T	C	1	F-L
19, 20	NS4B	6845	2250	A	G	25–27	E-G
18	NS4B	6850	2252	C	A	37	P-T
7	NS4B	6853	2253	A	G	10	K-E
18	NS4B	7252	2386	A	G	4	K-E
4, 5, 24	NS4B	7256	2387	C	A	90–97	T-N
15	NS5	7609	2505	A	C	1	K-Q
12	NS5	7966	2624	T	C	20	F-L
27	NS5	8107	2671	A	G	1	I-V
20	NS5	8423	2776	G	A	100	R-K
20	NS5	8500	2802	A	G	1	T-A
8	NS5	8731	2879	A	G	13	K-E
27	NS5	8888	2931	A	G	1	N-S
18	NS5	8947	2951	A	G	33	K-E
2	NS5	9157	3021	A	G	5	M-V
21, 20, 27	NS5	9210	3038	A	T	1–100	L-F
3	NS5	9503	3136	C	A	63	T-N
3	NS5	9518	3141	T	C	24	V-A

C—capsid; M—membrane; E—envelope; NS—non-structural.

## Data Availability

Not applicable.

## Supplementary Materials

The following are available online at https://www.mdpi.com/article/10.3390/v13081444/s1, Figure S1: Mean virus titer in serum, (a) of real time PCR positive in 49 patients, (b) of 27 patients with DENV isolates, Figure S2: The ration of frequencies of synonymous and non-synonymous muatation (nS/S) among structural and non-structural genes.
