# Supplementary material for: An Outbreak of Dengue Virus Serotype 2 Cosmopolitan Genotype in Nepal, 2017
Source: Viruses. 2021 Jul 24;13(8):1444. doi: 10.3390/v13081444 (PMC8402744; doi:10.3390/v13081444)

Supplementary Figure S1. Mean Virus titer in serum, (a) of real time PCR positive in 49 patients , (b) of 27 patients with DENV isolates.

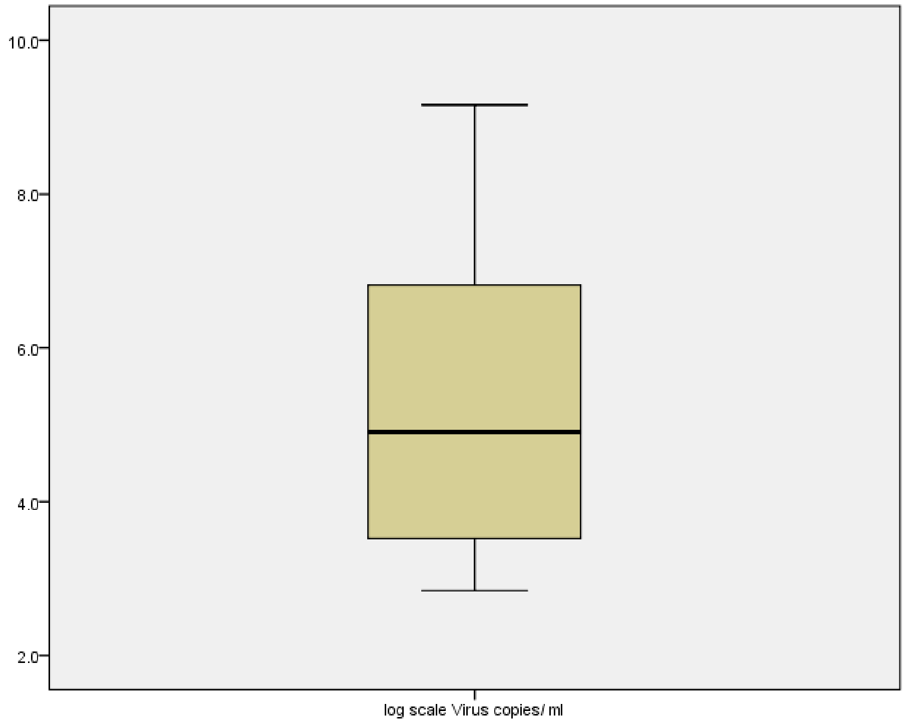


(a)

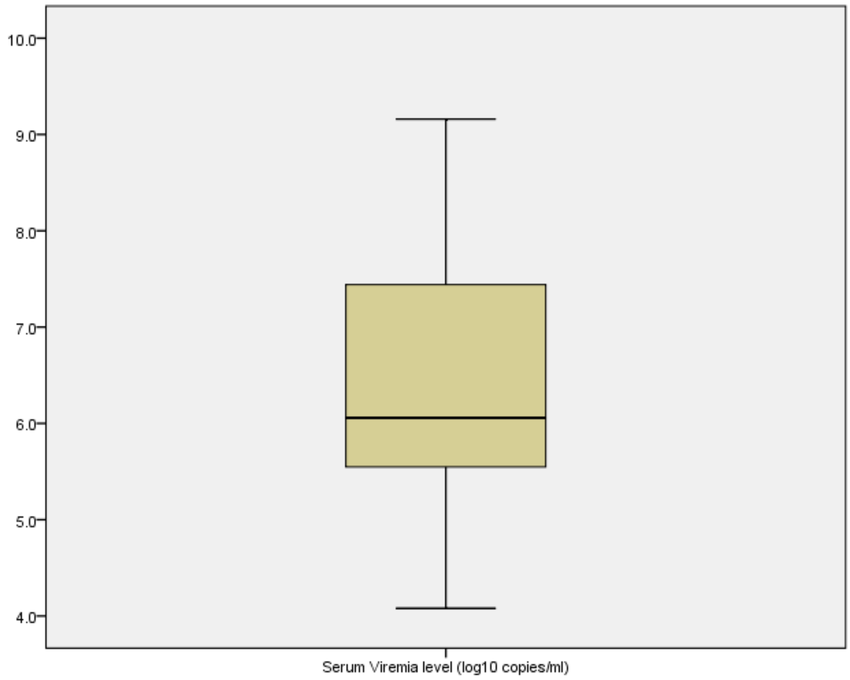


(b)

Supplementary Figure S2. The ration of frequencies of synonymous and nonsynonymous mutation (n S/S) among structural and non-structural genes.

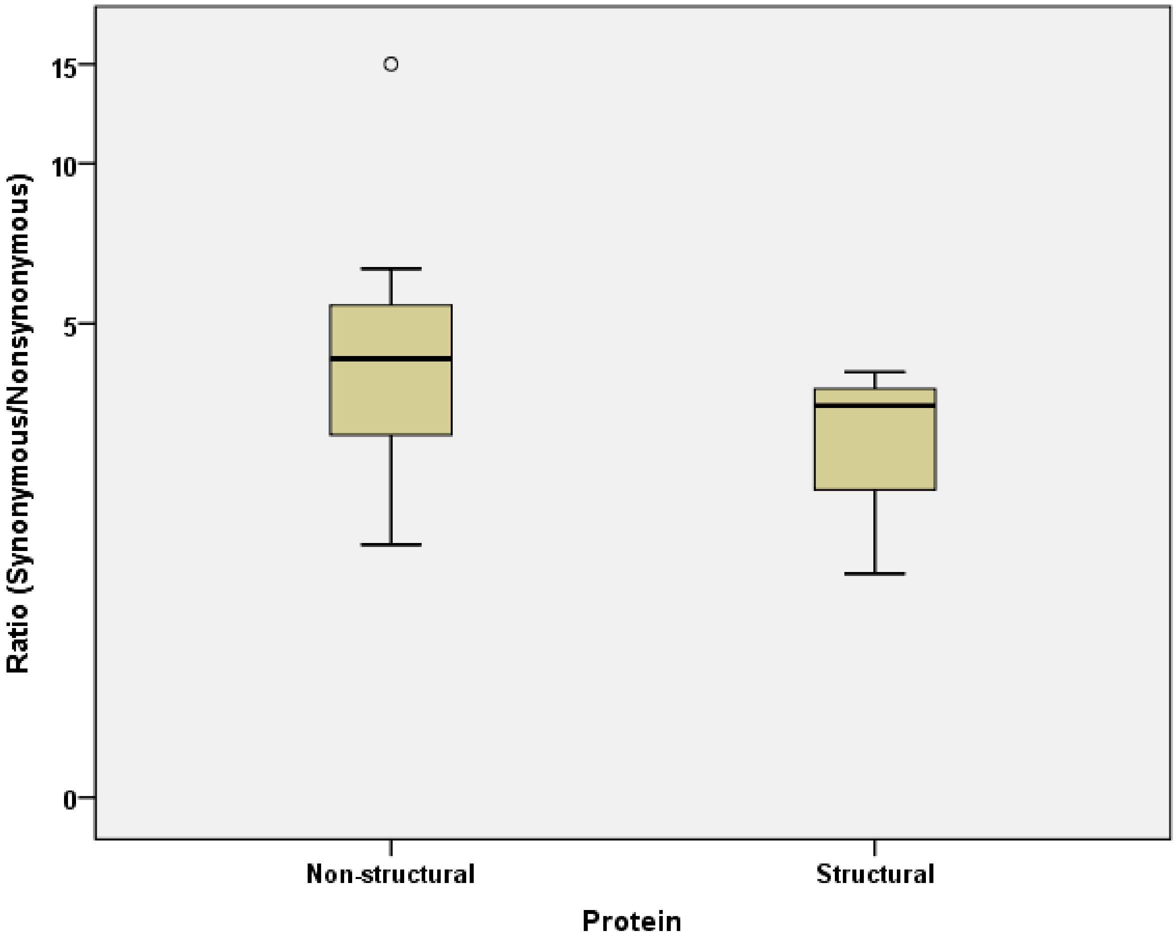

Supplement: Supplementary file 1 [file viruses-13-01444-s001.zip › viruses-1279289-supplementary.pdf]
